# Ototoxicity of Triclosan: A Rat Model Study

**DOI:** 10.7759/cureus.32189

**Published:** 2022-12-04

**Authors:** Çağlar Günebakan, Orhan K Kahveci, Selçuk Kuzu, Emine H Kandır

**Affiliations:** 1 Otolaryngology - Head and Neck Surgery, Afyonkarahisar Health Sciences University, Afyonkarahisar, TUR; 2 Faculty of Veterinary Medicine, Department of Wildlife and Ecology, Afyon Kocatepe University, Afyonkarahisar, TUR

**Keywords:** dose-dependant, ototoxicity, rat model, triclosan, hearing loss

## Abstract

Objective: Triclosan is utilized as an antibacterial factor in many industrial products. Although there are many toxic features of triclosan in the literature, there is no study on the effect of triclosan on hearing. The purpose of this study is to determine the effect of triclosan on hearing in rats.

Methods: In this prospective, experimental animal study, 40 healthy Sprague-Dawley rats with normal response to the distortion-product otoacoustic emission (DPOAE) measurements were divided into four groups. Group 1, as the control group, was given only corn oil, group 2 was given 5 mg/kg triclosan dissolved in corn oil, group 3 was given 10 mg/kg triclosan dissolved in corn oil, and group 4 was given 100 mg/kg triclosan dissolved in corn oil; triclosan and corn oil were administered by oral gavage to all groups.

Results: In our study, low-dose triclosan did not cause hearing loss, but hearing loss was observed in the group that was given high-dose triclosan (100 mg/kg).

Conclusion: These findings suggest that triclosan causes hearing loss in rats. This issue should be investigated further to avoid triclosan ototoxicity in humans.

## Introduction

Today, it is known that many drugs and chemicals have ototoxic effects. One of the chemicals we think to be potentially ototoxic is triclosan. Because of its antimicrobial properties, triclosan is a chemical substance that is widely used in many commercial products, such as soap, deodorant, and toothpaste, and is dangerous for human and animal health, with potentially toxic, carcinogenic, estrogenic, and neurotoxic effects [1,2].

This research intends to examine the negative impacts of triclosan on hearing within rats and to provide the necessary precautions for people using triclosan-containing products, which are widely used in different industries.

## Materials and methods

This study was carried out on 40 Sprague-Dawley rats produced at Afyon Kocatepe University Laboratory of Experimental Animals of the Faculty of Veterinary Medicine. The external ear canals and tympanic membranes of all rats were evaluated by otoscopic examination. Before the study, all rats were anesthetized with intramuscular ketamine hydrochloride 45 mg/kg and Xylocaine 5 mg/kg, then distortion-product otoacoustic emission (DPOAE) was measured. Distortion product emissions were measured using the software "ILO 288 Echoport USB" (Otodynamics, Hatfield, United Kingdom) and "EZ-SCREEN Type 2" (version 6.21.0.00, Banner Engineering, Minneapolis, MN). Eight rats with no DPOAE response were excluded from the study. A total of 40 rats were divided into four groups. A total of 20 ears were assessed in each group. Subjects were kept in an environment of 12 hours of daylight and 12 hours of dark, at 25°C, with access to free food and water, and a background noise level of less than 50 dB. Triclosan was dissolved in corn oil and prepared for oral gavage (o.g.).

Study groups

Group 1 (Corn Oil Group)

In this group of 10 adult rats, only corn oil was administered at 1 cc (o.g.) once a day for 30 days. DPOAE measurements were performed before the study and on the 30th day of the study.

Group 2 (Corn Oil + 5 mg/kg Triclosan o.g. Group)

In this group of 10 adult rats, 5 mg/kg triclosan dissolved in corn oil only for 30 days was administered at 1 cc (o.g.) once a day. DPOAE measurements were performed before the study and on the 30th day of the study.

Group 3 (Corn Oil + 10 mg/kg Triclosan o.g. Group)

In this group of 10 adult rats, 10 mg/kg triclosan dissolved in corn oil only for 30 days was administered at 1 cc (o.g.) once a day. DPOAE measurements were performed before the study and on the 30th day of the study.

Group 4 (Corn Oil + 100 mg/kg Triclosan o.g. Group)

In this group of 10 adult rats, 100 mg/kg triclosan dissolved in corn oil only for 30 days was administered at 1 cc (o.g.) once a day. DPOAE measurements were performed before the study and on the 30th day of the study.

Statistical analysis

Data analysis was performed using IBM SPSS Statistics version 22 (IBM Corp., Armonk, NY). Descriptive statistical methods (mean and standard deviation) as well as Wilcoxon paired two-sample tests were used to evaluate the study data. Significance was accepted at p < 0.05.

## Results

The comparison of DPOAE values of the corn oil group (group 1) before and on the 30th day of the study is shown in Table 1. There was no statistically significant difference between 1000 Hz, 1500 Hz, 2000 Hz, 3000 Hz, 4000 Hz, 6000 Hz, and 8000 Hz when the distortion-product (DP) values obtained before and after the 30th day of the study were compared (p > 0.05).

**Table 1:** Comparison of the DP values of the corn oil group before the study and on the 30th day of the study DP: distortion-product.

Frequency	Pre-drug average (standard deviation)	Post-drug average (standard deviation)	P-value
1000 Hz	-5.60 (7.70)	-6.20 (5.55)	0.310
1500 Hz	-5.78 (4.60)	-6.40 (8.68)	0.120
2000 Hz	-2.13 (11.32)	7.08 (10.55)	0.173
3000 Hz	5.86 (8.83)	7.56 (13.97)	0.515
4000 Hz	11.70 (11.81)	12.71 (15.58)	0.767
6000 Hz	27.06 (14.80)	25.56 (17.55)	0.678
8000 Hz	20.66 (17.78)	15.97 (16.83)	0.678

The DPOAE values of the corn oil + 5 mg/kg triclosan o.g. group (group 2) before the study and on the 30th day of the study are shown in Table 2. There was no statistically significant difference between 1000 Hz, 1500 Hz, 2000 Hz, 3000 Hz, 4000 Hz, 6000 Hz, and 8000 Hz when the DP values obtained before and after the 30th day of the study were compared (p > 0.05).

**Table 2:** Comparison of DP values of corn oil + 5 mg/kg triclosan o.g. group (group 2) before the study and on the 30th day of the study DP: distortion-product.

Frequency	Pre-drug average (standard deviation)	Post-drug average (standard deviation)	P-value
1000 Hz	-3.38 (7.98)	-1.88 (11.74)	0.065
1500 Hz	-2.68 (7.47)	1.02 (12.54)	0.343
2000 Hz	4.86 (8.23)	1.79 (13.82)	0.678
3000 Hz	7.64 (9.94)	4.57 (13.78)	0.374
4000 Hz	14.04 (9.06)	13.03 (12.36)	0.767
6000 Hz	27.84 (16.74)	29.62 (20.30)	0.515
8000 Hz	22.78 (15.61)	26.35 (19.50)	0.314

The DPOAE values of the corn oil + 10 mg/kg triclosan o.g. group (group 3) before the study and on the 30th day of the study are shown in Table 3. There was no statistically significant difference between 1000 Hz, 1500 Hz, 2000 Hz, 3000 Hz, 4000 Hz, 6000 Hz, and 8000 Hz when the DP values obtained before and after the 30th day of the study were compared (p > 0.05).

**Table 3:** Comparison of DP values of corn oil + 10 mg/kg triclosan o.g. group (group 3) before the study and on the 30th day of the study DP: distortion-product.

Frequency	Pre-drug average (standard deviation)	Post-drug average (standard deviation)	P-value
1000 Hz	-5.75 (8.25)	-4.10 (15.10)	0.767
1500 Hz	-3.02 (10.45)	-1.60 (12.55)	0.678
2000 Hz	1.95 (10.37)	-3.51 (16.27)	0.173
3000 Hz	4.63 (9.07)	1.49 (13.56)	0.678
4000 Hz	11.17 (10.84)	6.24 (15.81)	0.214
6000 Hz	20.72 (20.02)	21.56 (20.22)	0.779
8000 Hz	13.37 (18.29)	16.36 (19.49)	0.441

The DPOAE values of the corn oil + 100 mg/kg triclosan o.g. group (group 4) before the study and on the 30th day of the study are shown in Table 4. When the DP values obtained before and after the 30th day of the study were compared, there was no statistically significant difference in 1000 Hz, 1500 Hz, 6000 Hz, and 8000 Hz (p > 0.05), but statistically, a significant difference was found in 2000 Hz, 3000 Hz, and 4000 Hz (p < 0.05).

**Table 4:** Comparison of DP values of corn oil + 100 mg/kg triclosan o.g. group (group 4) before the study and on the 30th day of the study DP: distortion-product.

Frequency	Pre-drug average (standard deviation)	Post-drug average (standard deviation)	P-value
1000 Hz	-4.81 (7.77)	-3.34 (16.31)	0.285
1500 Hz	-1.43 (9.50)	-1.22 (15.57)	0.646
2000 Hz	3.28 (12.64)	-0.84 (18.09)	0.026
3000 Hz	6.99 (11.10)	1.53 (13.68)	0.037
4000 Hz	11.51 (12.27)	6.53 (17.86)	0.047
6000 Hz	24.9 (18.30)	26.48 (19.81)	0.386
8000 Hz	14.91 (20.91)	16.6 (20.54)	0.333

## Discussion

Triclosan (5-chloro-2-(2,4-dichlorophenoxy)phenol) is a broad-spectrum antimicrobial agent effective against gram-positive and gram-negative bacteria, some viruses, and fungi [3]. Due to its antimicrobial properties, triclosan has been widely used in various personal care products such as soap, shampoo, skin creams, toothpaste, deodorants, and cosmetic products. Besides, triclosan is present in many household items such as plastic kitchen utensils, amusement gadgets, footwear, dolls, and textiles [4,5]. The increasing fashionableness of antibacterial user goods has expanded the use of triclosan, particularly in liquid hand cleansers [6].

As a result of the widespread use and high stability of triclosan, it has been widely identified in wastewater, natural water, and drinking water [3-7]. Because of its lipophilic properties, triclosan has been detected in aquatic products and food samples such as salmon and even in breast milk, serum, and urine specimens [8]. Moreover, triclosan has been seen to remain in the environment for a long time, particularly under anaerobic conditions [9]. The most likely routes of human exposure to triclosan are swallowing and absorption through the skin [10].

The antiseptic activity of triclosan is due to its ability to block the synthesis of fatty acids by inhibiting the enoyl-acyl carrier protein reductase enzyme, which catalyzes an important step in many bacterial and fungal membranes [11]. However, the safety of triclosan in environmental and human health issues is questioned [12]. Although companies that produce triclosan-containing products claim the substance to be safe, the United States Environmental Protection Agency listed triclosan as a substance with unfavorable effects [13]. Besides, triclosan is easily transformed into chlorinated dibenzo-p-dioxins and chlorophenols by heat and ultraviolet radiation, which can be harmful to biological systems [14,15]. It has been reported in the literature that products using triclosan may have stronger side effects than triclosan itself [16]. Recently, triclosan has been included in the list of possible endocrine disruptors, since it has been shown to impair thyroid hormone homeostasis and possibly the reproductive axis. The phenolic structural relationship with nonsteroidal estrogen, diethylstilbestrol, and bisphenol A is also of concern [17].

The Japanese government has set the maximum amount of triclosan that can be used in cosmetics at 0.1%. The amount of triclosan allowed in oral care products in Canada is 0.03% and in cosmetic products is 0.3%. According to an Australian government report in 2009, triclosan has been identified as irritating to the eyes, respiratory system, and skin, and toxic to inhalation [18]. Recent studies have shown that the antimicrobial effects of triclosan are not so different from other soaps used in daily life. Since 2015, the Food and Drug Administration (FDA) has banned the use of triclosan in body shampoos and soaps [19].

Studies on the effects of triclosan on human well-being are usually carried out with experimental animals [20,21]. During contact with products containing triclosan, triclosan is taken from the skin, nose, and mouth. Also, triclosan is received from food such as seafood as a result of the triclosan reaching the food chain by mixing with sea, lake, and groundwater [1]. As a result of a series of studies conducted in the United States, 36 nursing mothers who stated that they use excessive triclosan-containing personal care products have been found to have significant amounts of triclosan in their milk [1]. Studies have revealed that triclosan alters androgens in males and estrogen in females. Triclosan has been shown to change transportation linking the fetus and placenta in a pregnant lamb. This was stated to induce unnatural development. In a series of investigations on rabbits, it was reported that triclosan reduces sperm number in male rabbits, causes parenchyma harm to reproductive glands, and disrupts male hormones [1]. It is known that the thyroid has vital effects on development and metabolism. The thyroid hormone is highly effective in the development of fetuses and young children. Studies have shown that triclosan reduces thyroid hormone levels in rabbits and changes the metamorphosis time in frogs [1,21].

Many adverse effects of triclosan have been shown in animal experiments. In a study conducted by Lan et al., three groups of eight rats were given triclosan in the form of gavage dissolved in corn oil at doses of 10, 50, and 200 mg/kg and one group received only corn oil as the control group. Eight weeks of application showed a significant decrease in sperm production in the group receiving 200 mg/kg triclosan. In conclusion, when the kinetic parameters described in the study and histopathological changes in the epididymis were taken into consideration, they concluded that triclosan could induce epididymal damage due to epididymal accumulation. Besides, researchers have reported that triclosan also stimulates sperm toxicity, leading to abnormal sperm morphology and lower sperm production [22].

In a literature review by Rodricks et al. on the subacute and chronic toxicity of triclosan, studies on mammalian kinds including mice, rats, hamsters, and baboons were evaluated. For systemic toxicity, except for endocrine deterioration, the impacts of triclosan are mainly limited to changes in the liver and kidneys. Triclosan caused changes in liver weight, liver enzymes, and liver hypertrophy, resulting in increased size and number of peroxisomes. Renal toxicity in rodents has been demonstrated by renal inflammation and tubular regeneration [23].

In a literature review by Rodricks et al., genotoxicity and mutagenicity studies using classic prokaryotic and eukaryotic systems related to triclosan were examined. Current evidence has led the authors to assume that triclosan is not genotoxic or mutagenic [23]. However, some studies suggest that triclosan may be genotoxic in some organisms and/or cell types [24]. Data from studies using conventional analysis methods means that triclosan is not genotoxic, mutagenic, or carcinogenic. However, there is some evidence that triclosan can induce genotoxicity in non-mammals, including algae and bivalves. Due to the limited number of studies dealing with the genotoxicity, mutagenesis, and carcinogenicity of triclosan in non-plant-derived systems, it is difficult to say with certainty that triclosan does not have the potential to damage the gene [23].

Triclosan’s structural similarity to estrogenic and androgenic structures such as polychlorinated biphenyls (PCBs), polybrominated diphenyl ethers (PBDEs), bisphenol A, and thyroid hormones suggest that triclosan may be effective in endocrine degradation using the structure-activity relationship [24-26]. Various studies have shown that triclosan is capable of affecting endocrine function in various species. Since triclosan is detected in human plasma, breast milk, urine, and water, and large amounts of triclosan are used regularly, it is thought that the possible endocrine effect of triclosan may be important for human health [27].

In some studies, it has been shown that triclosan disrupts lipid synthesis and metabolism. Triclosan binds and inactivates the enoyl reductase domain of type II fatty acid synthase in bacteria as the basis of its antibacterial properties [25]. Thus, the inhibitory effects of triclosan against fatty acid synthase and other lipid homeostasis components may have adverse effects on the lipid-rich central nervous system, but this hypothesis has not yet been proven [23].

In a study conducted on rats, triclosan was administered at doses of 0, 100, 300, 1000, and 2000 mg/kg/day for 14 days, and neurotoxicity was investigated. At a dose of 300 mg/kg/day, it was observed in the rats that mild movement inhibition decreased muscle tone, polydipsia, and polyuria. A 1000 mg/kg/day dose had more prominent findings. No changes in brain weight or histopathological findings were observed at any dose level tested. Peripheral nerve changes were not observed at any dose level tested. Current observations show that triclosan may cause adverse effects on central nervous system functions, primarily through the induction of apoptosis and oxidative stress [25]. Similarly, in our study, triclosan does not cause hearing loss in low doses, but high levels of hearing loss suggest that this substance may be the result of accumulation in the inner ear or neural tissues. In other words, triclosan given at high doses may lead to faster accumulation and may cause hearing loss. To prove this hypothesis, further histological studies should be performed on the inner ear and neural structures.

Tamura et al. suggested that triclosan increased intracellular zinc content by reducing thiol content in mouse cells at sublethal doses, which in turn caused cytotoxic effects by causing oxidative stress [26]. Another mechanism held responsible for cellular toxicity is the mechanism proposed by Kawanai. Accordingly, triclosan induces membrane hyperpolarization by increasing the concentration of intracellular Ca (2+) that activates Ca (2+)-dependent K (+) channels. The change in the membrane potential of lymphocytes affects cellular immune functions [27]. Considering the role of calcium channels in hearing, it can be suggested that the ototoxic effect of triclosan can be realized by its effect on calcium channels.

Studies have also emphasized that DPOAE is an effective method for detecting outer hair cell damage and is a sensitive method for assessing hearing loss even when abnormal hearing-related symptoms are present, but the audiogram is normal [28]. The production site of otoacoustic emissions (OAEs) is the outer shaky hairy cells. Due to hypoxia, ototoxic drugs, and acoustic trauma, the destruction of external hairy cells occurs and the production of OAEs is prevented [29]. In our study, we used the DPOAE test to evaluate the loss of external hairy cell function due to possible ototoxicity caused by triclosan.

In our study, when the DP values ​​obtained in the OAE measurements performed before and during the 30th day of the study were compared only in the corn oil group, no statistically significant difference was found in 1000 Hz, 1500 Hz, 2000 Hz, 3000 Hz, 4000 Hz, 6000 Hz, and 8000 Hz (p > 0.05). This shows that corn oil has no ototoxic effect.

In our study, for corn oil + 100 mg/kg triclosan oral gavage given before the study and on the 30th day of the study, there was no statistically significant difference (p > 0.05) in DP values ​​obtained in the OAE measurements compared to 1000 Hz, 1500 Hz, 6000 Hz, and 8000 Hz. A statistically significant difference was found at 2000 Hz, 3000 Hz, and 4000 Hz (p < 0.05). This shows that triclosan is ototoxic in rats.

Again in our study, for corn oil + 5 mg/kg triclosan and corn oil + 10 mg/kg triclosan given before the study and on the 30th day of the study compared to the DP values ​​obtained in OAE measurements at 1000 Hz, 1500 Hz, 2000 Hz, 3000 Hz, 4000 Hz, 6000 Hz, and 8000 Hz, there was no statistically significant difference (p > 0.05). Our study revealed triclosan has an ototoxic effect on rats with a 100 mg/kg dose. We believe further studies are needed to clarify the ototoxicity in humans.

## Conclusions

To summarize, triclosan is a very controversial chemical in recent times and it is found in many consumer goods. Although there are many toxic features of triclosan in the literature, there is no study on the effect of triclosan on hearing. In our study, low-dose triclosan did not cause hearing loss, but hearing loss was observed in the group that was given high-dose triclosan (100 mg/kg). These findings suggest that triclosan causes hearing loss in rats and further studies are needed to clarify the ototoxic effect in humans.

## References

[1] Orvos DR, Versteeg DJ, Inauen J, Capdevielle M, Rothenstein A, Cunningham V (2002). Aquatic toxicity of triclosan. Environ Toxicol Chem.

[2] (2009). Opinion on triclosan - antimicrobial resistance. https://ec.europa.eu/health/scientific_committees/consumer_safety/docs/sccs_o_023.pdf.

[3] Welsch TT, Gillock ET (2011). Triclosan-resistant bacteria isolated from feedlot and residential soils. J Environ Sci Health A Tox Hazard Subst Environ Eng.

[4] Gangadharan Puthiya Veetil P, Vijaya Nadaraja A, Bhasi A, Khan S, Bhaskaran K (2012). Degradation of triclosan under aerobic, anoxic, and anaerobic conditions. Appl Biochem Biotechnol.

[5] von der Ohe PC, Schmitt-Jansen M, Slobodnik J, Brack W (2012). Triclosan—the forgotten priority substance?. Environ Sci Pollut Res Int.

[6] Stoker TE, Gibson EK, Zorrilla LM (2010). Triclosan exposure modulates estrogen-dependent responses in the female Wistar rat. Toxicol Sci.

[7] Perez AL, De Sylor MA, Slocombe AJ, Lew MG, Unice KM, Donovan EP (2013). Triclosan occurrence in freshwater systems in the United States (1999-2012): a meta-analysis. Environ Toxicol Chem.

[8] Dann AB, Hontela A (2011). Triclosan: environmental exposure, toxicity and mechanisms of action. J Appl Toxicol.

[9] Chalew TE, Halden RU (2009). Environmental exposure of aquatic and terrestrial biota to triclosan and triclocarban. J Am Water Works Assoc.

[10] Clayton EM, Todd M, Dowd JB, Aiello AE (2011). The impact of bisphenol A and triclosan on immune parameters in the U.S. population, NHANES 2003-2006. Environ Health Perspect.

[11] Prasanth M (2011). Antimicrobial efficacy of different toothpastes and mouthrinses: an in vitro study. Dent Res J (Isfahan).

[12] Gonzalo-Lumbreras R, Sanz-Landaluze J, Guinea J, Cámara C (2012). Miniaturized extraction methods of triclosan from aqueous and fish roe samples. Bioconcentration studies in zebrafish larvae (Danio rerio). Anal Bioanal Chem.

[13] Rodríguez PE, Sanchez MS (2010). Maternal exposure to triclosan impairs thyroid homeostasis and female pubertal development in Wistar rat offspring. J Toxicol Environ Health A.

[14] Kumar V, Chakraborty A, Kural MR, Roy P (2009). Alteration of testicular steroidogenesis and histopathology of reproductive system in male rats treated with triclosan. Reprod Toxicol.

[15] Kliegman S, Eustis SN, Arnold WA, McNeill K (2013). Experimental and theoretical insights into the involvement of radicals in triclosan phototransformation. Environ Sci Technol.

[16] Sankoda K, Matsuo H, Ito M, Nomiyama K, Arizono K, Shinohara R (2011). Identification of triclosan intermediates produced by oxidative degradation using TiO2 in pure water and their endocrine disrupting activities. Bull Environ Contam Toxicol.

[17] Ahn KC, Zhao B, Chen J (2008). In vitro biologic activities of the antimicrobials triclocarban, its analogs, and triclosan in bioassay screens: receptor-based bioassay screens. Environ Health Perspect.

[18] (2009). Australian Government Department of Health and Ageing. Triclosan. https://nla.gov.au/nla.cat-vn4610392.

[19] Kim SA, Moon H, Lee K, Rhee MS (2015). Bactericidal effects of triclosan in soap both in vitro and in vivo. J Antimicrob Chemother.

[20] (2016). FDA issues final rule on safety and effectiveness of antibacterial soaps. https://www.fda.gov/NewsEvents/Newsroom/PressAnnouncements/ucm517478.htm.

[21] Orhan M, Kut D, Güneşoğlu C (2007). Use of triclosan as antibacterial agent in textiles. Indian J Fibre Text Res.

[22] Lan Z, Hyung Kim T, Shun Bi K, Hui Chen X, Sik Kim H (2015). Triclosan exhibits a tendency to accumulate in the epididymis and shows sperm toxicity in male Sprague-Dawley rats. Environ Toxicol.

[23] Rodricks JV, Swenberg JA, Borzelleca JF, Maronpot RR, Shipp AM (2010). Triclosan: a critical review of the experimental data and development of margins of safety for consumer products. Crit Rev Toxicol.

[24] McMurry LM, Oethinger M, Levy SB (1998). Triclosan targets lipid synthesis. Nature.

[25] Ruszkiewicz JA, Li S, Rodriguez MB, Aschner M (2017). Is triclosan a neurotoxic agent?. J Toxicol Environ Health B Crit Rev.

[26] Tamura I, Kanbara Y, Saito M, Horimoto K, Satoh M, Yamamoto H, Oyama Y (2012). Triclosan, an antibacterial agent, increases intracellular Zn(2+) concentration in rat thymocytes: its relation to oxidative stress. Chemosphere.

[27] Bishop GM, Dringen R, Robinson SR (2007). Zinc stimulates the production of toxic reactive oxygen species (ROS) and inhibits glutathione reductase in astrocytes. Free Radic Biol Med.

[28] Avan P, Bonfils P (2005). Distortion-product otoacoustic emission spectra and high-resolution audiometry in noise-induced hearing loss. Hear Res.

[29] Probst R, Lonsbury-Martin BL, Martin GK (1991). A review of otoacoustic emissions. J Acoust Soc Am.

